# The Prognostic Value of Retroperitoneal Lymphadenectomy in Apparent Stage IA Endometrial Endometrioid Cancer

**DOI:** 10.3389/fonc.2020.618499

**Published:** 2021-02-16

**Authors:** Zhao Liu, Jinghe Lang, Ming Wu, Lei Li

**Affiliations:** ^1^ Department of Obstetrics and Gynecology, Peking Union Medical College Hospital, Beijing, China; ^2^ Department of Obstetrics and Gynecology, Beijing Aerospace General Hospital, Beijing, China

**Keywords:** endometrial carcinoma, endometrioid subtype, lymph node metastasis, disease-free survival, overall survival

## Abstract

**Study design:**

Retrospective cohort study.

**Introduction:**

Debates remain regarding the role of lymphadenectomy in patients with apparent stage IA endometrial cancer, especially subtypes with a favorable prognosis. This study aimed to explore the prognostic value of staging surgeries in apparent stage IA endometrial endometrioid cancer patients in a retrospective cohort study.

**Methods:**

Cases from June 1, 2010 to June 1, 2017 were reviewed in patients with pathologically confirmed endometrial endometrioid carcinoma limited to <1/2 of the myometrium, without extrauterine metastasis on preoperative evaluation and during surgical inspection. Survival outcomes were compared between patients with and without lymphadenectomy and between patients with and without metastasis to lymph nodes.

**Results:**

In total, 1,312 eligible patients were included, among which 836 underwent staging surgeries and 476 underwent simple hysterectomy. Twenty-eight patients were found with metastasis to retroperitoneal lymph nodes. After a median follow-up of 57.4 months, lost to follow-up, recurrence, death, and cancer-specific death occurred in 28, 39, 24, and 16 patients, respectively. In a univariate analysis, lymphadenectomy of the pelvis with or without para-aortic lymph nodes had no significant impact on disease-free survival, overall survival or cancer-specific overall survival (*p* values >0.05). However, after adjusting for important baseline risk factors [menopausal status, tumor differentiation, maximum diameter and location, lymph-vascular space invasion (LVSI) status, and postoperative adjuvant therapy), lymphadenectomy resulted in significantly improved survival outcomes (*p* values <0.05). Menopause (odds ratio [OR] 4.7, 95% confidence interval [CI] 1.3–16.4, *p*=0.015), tumor diameter larger than 2 cm (OR 4.6, 95% CI 1.3–16.0, *p*=0.016), grade 3 tumors (OR 3.0, 95% CI 1.0–8.5, *p*=0.042), positive LVSI (OR 8.7, 95% CI 3.7–20.4, *p*<0.001) and lower uterine segment involvement (OR 3.1, 95% CI 1.4–7.2, *p*=0.007) had more extrauterine metastases.

**Conclusion:**

In cases of apparent stage IA endometrioid endometrial carcinoma, staging surgeries should be considered in patients with larger, higher grade tumors, positive LVSI, or lower uterine segment involvement.

## Introduction

In the United States and in China, uterine cancer is estimated as the fourth and the ninth most common cancer in terms of new cases, and the sixth and the 10th most common cause of cancer death, respectively (1, 2). Endometrial cancer is often diagnosed at an early stage, as it frequently involves abnormal vaginal bleeding (3). In previous reports, no evidence of benefit exists in terms of overall or recurrence-free survival for pelvic lymphadenectomy in women with early stage endometrial cancer (4, 5), although systematic pelvic lymphadenectomy significantly improves the surgical staging (5). However, these findings are fairly controversial (6–8). These studies all had their limitations, including patient selection, extent of lymph node dissection, and application of postoperative adjuvant therapy (9, 10). Lymphadenectomy is believed to identify patients requiring adjuvant therapy (11). The National Comprehensive Cancer Network (NCCN) panel even recommends that lymphadenectomy should be performed in select high-risk patients with endometrial cancer with para-aortic lymphadenectomy (12, 13).

As debates remain regarding the role of lymphadenectomy in patients with apparent stage IA endometrial cancer, especially those with the endometrioid subtype, we performed a retrospective study to explore the prevalence of metastasis to retroperitoneal lymph nodes and the prognostic value of staging surgeries in patients with apparent stage IA endometrial cancer. The primary objectives were to compare the 5-year rates of disease-free survival (DFS), overall survival (OS) and cancer-specific OS between patients with and without lymphadenectomy and between patients with and without metastasis to lymph nodes. The secondary objectives were to examine patterns and risk factors of lymph node metastasis.

## Methods

### Ethical Approval

The Institutional Review Board from the study center approved the study (No. ZS-1428). All patients provided written consent before treatment. Consent for participation from the next of kin may have been required for the study. The registration number is NCT03291275 (*clinicaltrials.gov*, registered on September 25, 2017). All procedures performed in the study involving human participants were in accordance with the ethical standards of the institutional and/or national research committee and with the 1964 Declaration of Helsinki and its later amendments or comparable ethical standards.

### Study Design

This study was a retrospective cohort study implemented in a tertiary teaching hospital. Apparent stage IA endometrial cancer in our study was defined as a tumor limited within <1/2 of the endometrium, without extrauterine metastasis on the preoperative imaging evaluation and in the gross inspection during staging surgeries or simple hysterectomy. All eligible patients were classified into groups with and without retroperitoneal lymphadenectomy and groups with and without metastasis to lymph nodes. The sites of lymphadenectomy and metastasis were further classified into pelvic and para-aortic lymph nodes (PLN and PALN, respectively).

### Patient Enrollment

Detailed surgical and pathological data were collected by searching and reviewing electronic medical records from June 1, 2010 to June 1, 2017 at the study center. No authors had access to information that could identify individual participants during or after data collection, except for the corresponding author, who was in charge of the data collection. Information on fertility, smoking status and metabolic diseases (hypertension, hyperlipemia, diabetes, and obesity/overweight) was recorded. The inclusion criteria consisted of the following: primary endometrial cancer of the endometrioid subtype confirmed before surgery; preoperative imaging assessment and intraoperative gross inspection of the uterus identified as <1/2 myometrium invasion without extrauterine metastasis; and detailed clinicopathological records to be traced. A patient was excluded if she: had no pathological evaluation before the surgeries or if the evaluation suggested a non-endometrioid subtype before or after the surgeries; had no preoperative imaging; had suspicious deep myometrium invasion or extrauterine metastasis before or during surgery; or had actual deep myometrium invasion.

### Preoperative Imaging and Intraoperative Inspection

Information regarding preoperative imaging and intraoperative inspection was retrospectively extracted from medical and surgical records. The imaging evaluation included ultrasound, computed tomography (CT), or magnetic resonance imaging (MRI). Data on the myometrium invasion status were the most essential. However, any suspicious metastasis to the peritoneum, omentum, adnexa, viscera, lymph nodes or thorax was also specifically checked in the surgical and pathological records.

### Interventions and Follow-Up

Patients underwent single hysterectomy or comprehensive staging, which included hysterectomy, bilateral salpingectomy or bilateral salpingoophorectomy, and retroperitoneal lymphadenectomy (resection of PLN with or without para-aortic PALN). The resection of PALN was further classified as below or above the level of the inferior mesenteric artery. The surgical selection was based on discussion and communication between physicians and patients, as well as the consideration of relevant risk factors. However, not all case reports revealed the definite reasons for staging surgeries. The surgeries were performed by laparoscopy or laparotomy. Protocols, regimens and cycles of chemotherapy were extracted from electronic records. All adjuvant therapies followed the relevant contemporary guidelines.

The pathological outcomes of all patients were comprehensively reviewed. The significant characteristics of pathological evaluation included maximum diameter, differentiation, lymph-vascular space invasion (LVSI), involvement of the lower uterine segment (14), numbers and metastasis of harvested lymph nodes, and results of peritoneal cytology. The tumor location was further divided into limitations to the endometrium and <1/2 myometrium invasion.

All patients were followed until February 1, 2019. Close follow-up according to a customized protocol was provided for all patients who visited an outpatient clinic every 3–4 months for the first year, every 6 months for the second and third years, and every year for the remainder of the follow-up time. Follow-up protocols consisted of physical examinations, cytology tests, and imaging evaluations. Imaging evaluations included pelvic sonography at each visit, and pelvic MRI and thoracoabdominal CT once a year. Recurrence was validated by physical examination, imaging examination, and/or biopsy. The recurrent sites were divided into the following categories: local (pelvic cavity and vagina), regional (abdominal cavity), and distant (outside the abdominal cavity). Mortality was confirmed by reviewing medical records and interviews by telephone and/or email. DFS was defined as the length of time that a patient lived after the major surgery without any signs or symptoms of endometrial cancer; OS was defined as the length of time that a patient was still alive after the diagnosis of endometrial cancer.

### Statistics

Comparisons of continuous variables were conducted with parametric methods if assumptions of normal distribution were confirmed. Nonnormally distributed variables and categorical data were compared among three groups using nonparametric tests. Survival curves were generated with the Kaplan–Meier method, and proportional hazards models were used to estimate the hazard ratios (HRs) and 95% confidence intervals (CIs) for the effect of systematic lymphadenectomy on DFS and OS. A multivariable analysis of DFS and OS was performed, with adjustments for important baseline risk factors (menopausal status, grade of differentiation, maximum diameter of the tumor, LVSI status, lower uterine segment involvement, tumor limited to the endometrium, and postoperative adjuvant therapy). A binary logistic regression model was performed for the analysis of risk factors for metastasis to lymph nodes with odds ratios (ORs) and 95% CI. Unless otherwise stated, all analyses were performed with a two-sided significance level of 0.05 and conducted using the software SPSS 22.0 (SPSS, Inc., Chicago, IL, USA).

## Results

### Patient Characteristics

**Figure 1:**
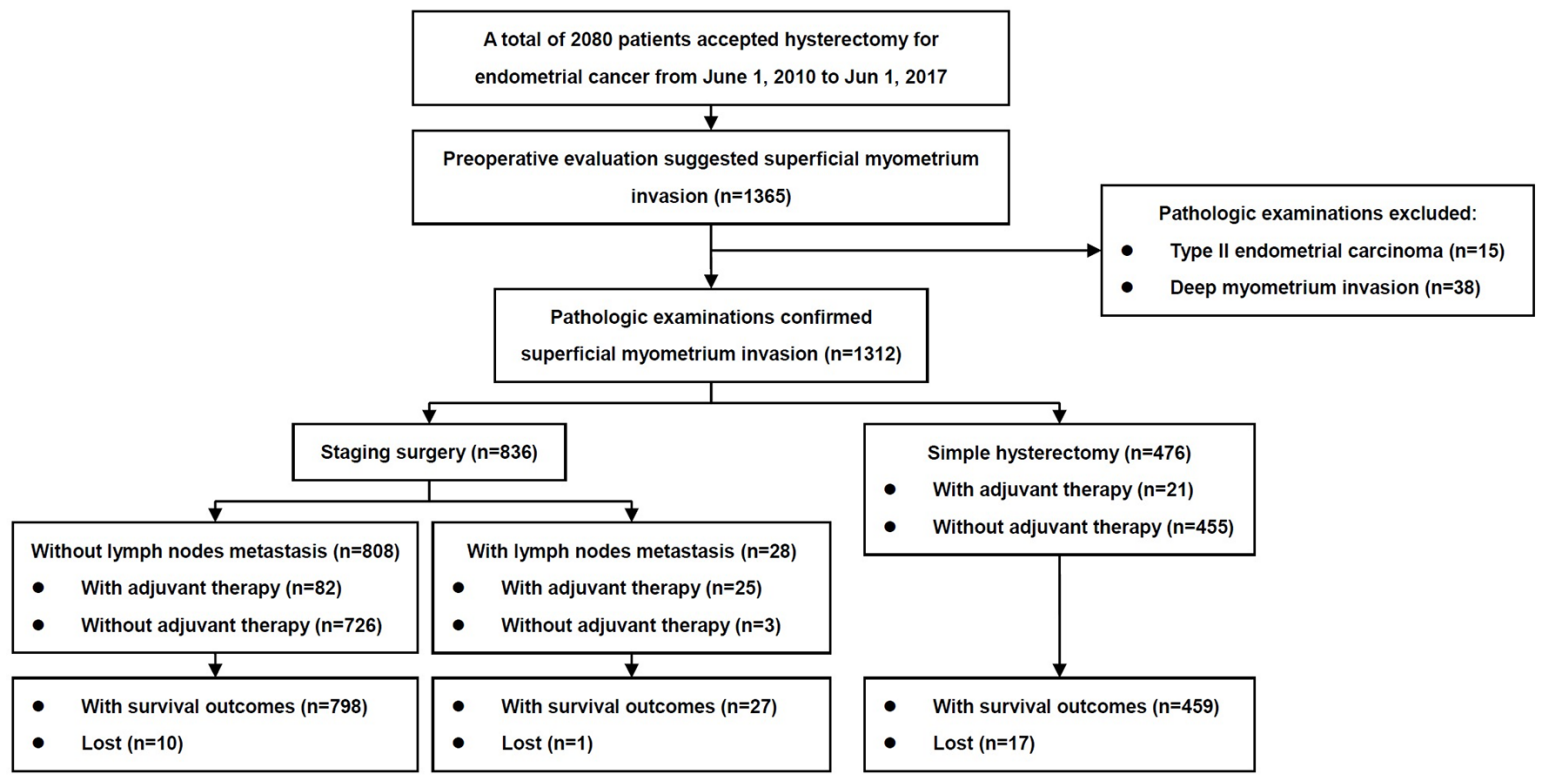
Flow diagram of the study.

The inclusion criteria of patients are shown in **Figure 1**. The baseline characteristics of the patients with and without metastasis to lymph nodes are summarized in **Table 1**. From June 1, 2010 to June 1, 2017, 1,365 patients with endometrial cancer underwent staged surgeries or simple hysterectomy by one of 26 physicians, and the final pathological examinations confirmed endometrial cancer. Fifteen patients with non-endometrioid carcinomas and 38 patients with deep myometrium invasion were excluded. In total, 1,312 cases were included in the study, with confirmed endometrioid endometrial carcinomas limited to <1/2 of the myometrium and with or without metastasis only to the lymph nodes. There were 836 cases (63.7%) of surgery staging and 476 cases (36.3%) of simple hysterectomy. Among patients with surgery staging, 28/836 (3.3%) were found to have lymph node metastasis.

**Table 1:** Epidemiological and clinicopathological characteristics of patients with and without metastasis to lymph nodes.

	Patients with no metastasis to lymph nodes of staging surgeries (n=808)	Patients with metastasis to lymph nodes of staging surgeries (n=28)	Patients with simple hysterectomy (n=476)	*p*
Age (year), mean ± SD	53.0 ± 9.1	55.3 ± 6.9	53.6 ± 10.9	0.322
Menopause, n (%)	513 (63.5%)	25 (89.3%)	303 (63.7%)	0.019
Metabolic disease, n (%)	302 (37.4%)	13 (46.4%)	183 (38.4%)	0.601
Diabetes	137 (17.0%)	4 (14.3%)	74 (15.5%)	0.769
Hypertension	240 (29.7%)	11 (39.3%)	151 (31.7%)	0.454
Hyperlipemia	31 (3.8%)	1 (3.6%)	20 (4.2%)	0.943
Obesity	30 (3.7%)	2 (7.1%)	17 (3.6%)	0.625
History of infertility, n (%)	13 (1.6%)	1 (3.6%)	6 (1.3%)	0.594
Smoking, n (%)	23 (2.8%)	2 (7.1%)	15 (3.2%)	0.424
Diagnostic methods, n (%)				<0.001
Dilation and curettage	417 (51.6%)	15 (53.6%)	212 (44.5%)	
Hysteroscopy	391 (48.4%)	13 (46.4%)	228 (47.9%)	
Accident finding	0 (0.0%)	0 (0.0%)	36 (7.6%)	
Surgical routs, n (%)				<0.001
Laparoscopy	602 (74.5%)	12 (42.9%)	370 (77.7%)	
Abdominal surgeries	206 (25.5%)	16 (57.1%)	106 (22.3%)	
PALN resection, n (%)				0.061
Not done	355 (43.9%)	6 (21.4%)	N/A	
Below the level of inferior mesenteric artery	6 (21.4%)	8 (28.6%)	N/A	
Above the level of inferior mesenteric artery	361 (43.2%)	14 (50.0%)	N/A	
Ovarian preservation, n (%)	14 (1.7%)	0 (0.0%)	26 (5.5%)	0.001
Peritoneal cytology, n (%)				<0.001
Not done	249 (30.8%)	8 (28.6%)	281 (59.0%)	
Done	559 (69.2%)	20 (71.4%)	195 (41.0%)	
Differential of endometrioid EC, n (%)				<0.001
Grade 1	506 (62.6%)	10 (35.7%)	387 (81.3%)	
Grade 2	248 (30.7%)	11 (39.9%)	78 (16.4%)	
Grade 3	54 (6.7%)	7 (25%)	11 (2.3%)	
Maximum diameter of the tumor (mm), mean ± SD	24.5 ± 19.3	37.7 ± 21.2	19.9 ± 15.9	<0.001
Tumor limited to the endometrium, n (%)	145 (17.9%)	1 (3.6%)	151 (31.7%)	<0.001
Positive LVSI, n (%)	54 (6.7%)	13 (46.4%)	15 (3.2%)	<0.001
Lower uterine involvement, n (%)	166 (20.5%)	14 (50.0%)	62 (13.0%)	<0.001
Positive peritoneal cytology, n (%)	18/559 (3.2%)	2/20 (10.0%)	4/195 (2.1%)	0.142
Harvested number of PLN, mean ± SD	24.0 ± 9.7	24.5 ± 10.2	N/A	0.772
Harvested number of PALN, mean ± SD	7.7 ± 5.8 (n=453)	7.7 ± 4.8 (n=22)	N/A	0.986
Post-operative adjuvant therapy, n (%)	82 (10.1%)	25 (89.3%)	21 (4.4%)	<0.001
Post-operative radiotherapy, n (%)	62 (7.7%)	17 (60.7%)	12 (2.5%)	<0.001
Post-operative chemotherapy, n (%)	35 (4.3%)	16 (57.1%)	10 (2.1%)	<0.001
Post-operative chemotherapy protocols, n (%)	n=35	n=16	n=10	0.517
Carboplatin+paclitaxel	29 (82.9%)	14 (87.5%)	7 (70.0%)	
Others	6 (17.1%)	2 (12.5%)	3 (30.0%)	
Post-operative chemotherapy cycles, mean ± SD	3.6 ± 1.7 (n=35)	6.0 ± 0.8 (n=16)	4.2 ± 2.0 (n=10)	<0.001
Recurrent sites, n (%)	n=19	n=3	n=17	0.520
Local (pelvic cavity and vagina)	7 (36.8%)	1 (33.3%)	11 (64.7%)	
Regional (abdominal cavity)	7 (36.8%)	1 (33.3%)	3 (17.6%)	
Distant (outside abdominal cavity)	5 (26.3%)	1 (33.3%)	3 (17.6%)	

EC, endometrial carcinoma; LVSI, lymph-vascular space invasion; N/A, not available; PALN, para-aortic lymph nodes; SD, standard deviation.

Lymphadenectomy of PALN was performed in 475/836 (56.8%) patients of staging surgeries. Sixty-six patients underwent sentinel lymph node mapping during the staged surgeries, and the distribution of negative findings (62 patients, 93.9%) and positive findings (4 patients, 6.1%) was similar to that in the general population.

For all patients with surgery staging, metastasis to lymph nodes, PLN and PALN occurred in 28/836 (3.3%), 23/836 (2.8%), and 11/476 (2.3%) patients, respectively. For 11 patients with PALN metastasis, three occurred below and eight above the level of the inferior mesenteric artery. For 28 patients with metastasis to lymph nodes, six only had resection of PLN, 17 had metastasis to both PLN and PALN, and five had metastasis only to PALN. For patients with and without PALN metastasis, there were no significant differences in the clinicopathological characteristics.

During the lymphadenectomy procedures, among 836 patients, the most common complication was heavy hemorrhage needing transfusion (five cases, 0.6%) and injury to the ureters (five cases, 0.9%), followed by injuries to obturator nerves (four cases, 0.5%), major vessels (three cases, 0.4%), and intestines (two cases, 0.2%). Only two cases were converted to open surgeries from laparoscopy. After staged surgeries, lymphocele was confirmed by imaging evaluation in 332 patients (39.7%), among which 12 patients had to be readmitted for lymphocele.

### Survival Outcomes

**Figure 2:**
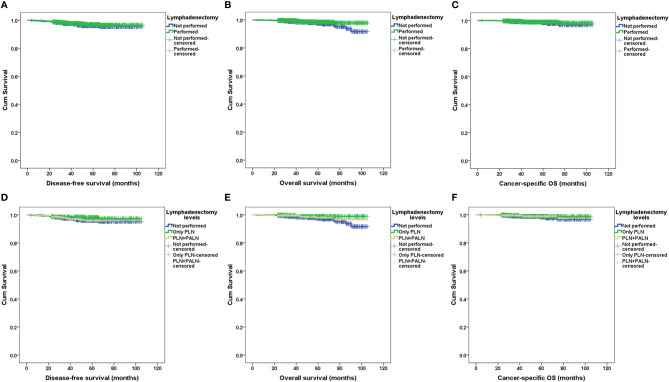
Survival outcomes between patients with and without retroperitoneal lymphadenectomy and various levels of lymphadenectomy by Kaplan–Meier analysis. DFS, disease-free survival; OS, overall survival; PALN, para-aortic lymph nodes; PLN, pelvic lymph nodes. **(A)** DFS in patients with and without lymphadenectomy. **(B)** OS in patients with and without lymphadenectomy. **(C)** Cancer-specific OS in patients with and without lymphadenectomy. **(D)** DFS in patients with various lymphadenectomy levels. **(E)** OS in patients with various lymphadenectomy levels. **(F)** Cancer-specific OS in patients with various lymphadenectomy levels.

**Figure 3:**
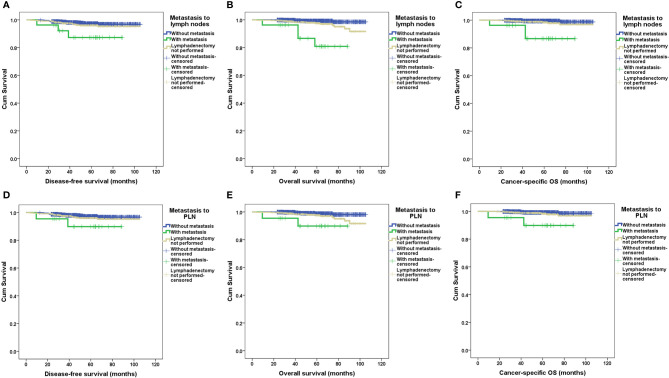
Survival outcomes between patients with and without metastasis to retroperitoneal lymph nodes by Kaplan–Meier analysis. DFS, disease-free survival; OS, overall survival; PALN, para-aortic lymph nodes; PLN, pelvic lymph nodes. **(A)** DFS in patients with and without metastasis to PLN and/or PALN. **(B)** OS in patients with and without metastasis to PLN and/or PALN. **(C)** Cancer-specific OS in patients with and without metastasis to PLN and/or PALN. **(D)** DFS in patients with and without metastasis to PLN. **(E)** OS in patients with and without metastasis to PLN. **(F)** Cancer-specific OS in patients with and without metastasis to PLN.

After a median follow-up of 57.4 months (range 3.8–105.4 months), 1,284 patients (97.9%) had definite outcomes of recurrence and survival. Recurrence and death occurred in 39 and 24 patients, respectively. Eight of 24 (33.3%) deaths were not due to endometrial cancer (**Supplementary Data Sheet 1**). For patients without metastasis to lymph nodes, with metastasis to lymph nodes, and with only simple hysterectomy, recurrence occurred in 19 (2.4%), three (11.1%), and 17 (3.7%) patients, respectively; death occurred in six (0.8%), four (14.8%), and 14 (3.1%) patients, respectively; relevant 5-year DFS rates were 97%, 87%, and 96% (p=0.021), respectively; relevant 5-year OS rates were 99%, 80%, and 98% (p<0.001), respectively; and relevant 5-year cancer-specific OS rates were 99%, 86%, and 98% (p<0.001), respectively. No significant differences existed in the recurrent sites. The survival outcomes of patients with and without lymphadenectomy and with and without metastasis to lymph nodes according to the Kaplan–Meier analysis are illustrated in **Figures 2** and **3**, respectively, and relevant HRs from the univariate and in multivariate analyses are listed in **Tables 2** and **3**, respectively.

**Table 2:** Hazard ratios (HRs) and 95% confidence intervals (95% CIs) by proportional hazards models for the effect of systematic lymphadenectomy on disease-free survival (DFS), overall survival (OS) and cancer-specific OS in the univariate analysis.

	DFS		OS		Cancer-specific OS	
	HR (95% CI)	*p*	HR (95% CI)	*p*	HR (95% CI)	*p*
Lymphadenectomy						
Not performed	Reference	–	Reference	–	Reference	–
Preformed	0.7 (0.4–1.4)	0.368	0.4 (0.2–1.0)	0.042	0.6 (0.2–1.6)	0.292
Lymphadenectomy levels			0.351	0.096		0.527
Not performed	Reference	–	Reference	–	Reference	–
Preformed only in PLN	0.5 (0.2–1.3)	0.158	0.3 (0.1–0.9)	0.041	0.5 (0.1–1.8)	0.278
Preformed in PLN and PALN	0.9 (0.5–1.8)	0.825	0.6 (0.2–1.4)	0.233	0.7 (0.2–2.1)	0.509
Metastasis to lymph nodes		0.034		<0.001		<0.001
Without metastasis	Reference	–	Reference	–	Reference	–
With metastasis	4.8 (1.4–16.2)	0.011	21.0 (5.9–74.9)	<0.001	17.8 (4.2–74.6)	<0.001
Lymphadenectomy not performed	1.5 (0.8–2.9)	0.225	3.7 (1.4–9.7)	<0.001	2.6 (0.9–8.0)	0.091
Metastasis to PLN		0.167		0.007		0.010
Without metastasis	Reference	–	Reference	–	Reference	–
With metastasis	3.6 (0.8–15.5)	0.082	9.1 (1.9–43.1)	0.005	11.6 (2.3–57.8)	0.003
Lymphadenectomy not performed	1.4 (0.8–2.7)	0.277	2.8 (1.2–6.7)	0.019	2.2 (0.8–6.3)	0.146
Metastasis to PALN in patients without PLN involved		0.063		<0.001		0.003
Without metastasis	Reference	–	Reference	–	Reference	–
With metastasis	8.7 (1.1–67.0)	0.038	76.2 (12.3–471.2)	<0.001	56.1 (4.9–637.9)	0.001
Lymphadenectomy not performed	0.7 (0.3–1.8)	0.449	1.1 (0.2–5.4)	0.914	1.7 (0.3–10.1)	0.567

PALN, para-aortic lymph nodes; PLN, pelvic lymph nodes.

**Table 3:** Hazard ratios (HRs) and 95% confidence intervals (95% CIs) by proportional hazards models for the effect of systematic lymphadenectomy on disease-free survival (DFS), overall survival (OS) and cancer-specific OS in the multivariate analysis.

	DFS		OS		Cancer-specific OS	
	HR (95% CI)	*p*	HR (95% CI)	*p*	HR (95% CI)	*p*
Lymphadenectomy						
Not performed	Reference	–	Reference	–	Reference	–
Preformed	0.4 (0.2–0.7)	0.004	0.2 (0.1–0.4)	<0.001	0.2 (0.1–1.0)	0.003
Lymphadenectomy levels		0.016		0.001		0.010
Not performed	Reference	–	Reference	–	Reference	–
Preformed only in PLN	0.4 (0.2–0.9)	0.035	0.2 (0.05–0.6)	0.006	0.2 (0.1–1.0)	0.042
Preformed in PLN and PALN	0.3 (0.2–0.8)	0.009	0.2 (0.06–0.5)	0.002	0.1 (0.03–0.5)	0.005
Metastasis to lymph nodes		0.016		<0.001		0.003
Without metastasis	Reference	–	Reference	–	Reference	–
With metastasis	1.4 (0.4–5.2)	0.649	8.3 (1.8–37.9)	0.006	7.2 (1.2–41.0)	0.027
Lymphadenectomy not performed	2.8 (1.4–5.8)	0.004	8.0 (2.7–23.3)	<0.001	7.4 (2.0–26.4)	0.002
Metastasis to PLN		0.017		0.001		0.008
Without metastasis	Reference	–	Reference	–	Reference	–
With metastasis	1.0 (0.2–4.7)	0.977	2.6 (0.5–15.0)	0.272	3.5 (0.6–22.5)	0.182
Lymphadenectomy not performed	2.8 (1.4–5.6)	0.005	6.4 (2.4–17.2)	<0.001	6.4 (1.9–21.7)	0.003
Metastasis to PALN in patients without PLN involved		0.681		0.029		0.211
Without metastasis	Reference	–	Reference	–	Reference	–
With metastasis	2.6 (0.3–24.0)	0.384	31.2 (2.5–394.3)	0.008	22.2 (0.6–852.3)	0.095
Lymphadenectomy not performed	1.0 (0.4–2.7)	0.977	1.7 (0.3–9.7)	0.526	2.4 (0.4–16.0)	0.351

PALN, para-aortic lymph nodes; PLN, pelvic lymph nodes.

In the univariate analysis, lymphadenectomy, either performed in PLN or in PLN plus PALN, had no significant impact on the DFS, OS, or cancer-specific OS (*p* values >0.05, **Table 2**). However, lymphadenectomy, either performed in PLN or PALN, significantly improved the survival outcomes after being adjusted for clinicopathological and therapeutic characteristics (*p* values <0.05, **Table 3**).

Obviously, metastasis to PLN, PALN, or PLN plus PALN caused significantly decreased DFS, OS and cancer-specific OS in either the univariate or multivariate analysis (*p* values <0.05, **Tables 2** and **3**). In the multivariate analysis, if lymphadenectomy was performed, metastasis to lymph nodes resulted in a similar recurrent risk compared with patients without metastasis to lymph nodes (*p* values <0.05), but resulted in a significantly higher risk of mortality (p values >0.05, **Table 3**). Patients with simple hysterectomy had an even higher risk of recurrence and mortality compared with patients with negative lymph node involvement (*p* values <0.05, **Table 3**).

### Risk Factors of Lymph Node Metastasis

For all patients with surgery staging, in the univariate analysis, tumor diameters, menopausal status, differentiation, tumor limited to the endometrium, LVSI and lower uterine segment involvement were risk factors for metastasis to lymph nodes. Based on these parameters, in a binary logistic regression model, factors of tumor diameters (p=0.048), menopausal status (p=0.017), differentiation (p=0.046), LVSI (p<0.001) and lower uterine segment involvement (p=0.020) were independent risk factors for metastasis to lymph nodes. We further categorized the tumor diameter into <20 mm versus >20 mm and the differentiation into grades 1 and 2 versus grade 3 and discovered that menopausal patients (OR 4.7, 95% CI 1.3–16.4, p=0.015) with larger (OR 4.6, 95% CI 1.3–16.0, p=0.016), higher grade tumors (OR 3.0, 95% CI 1.0–8.5, p=0.042), positive LVSI (OR 8.7, 95% CI 3.7–20.4, p<0.001), and lower uterine segment involvement (OR 3.1, 95% CI 1.4–7.2, p=0.007) had more extrauterine metastases.

## Discussion

Our study data suggest that for apparent stage IA endometrial carcinomas, even those of the endometrioid subtype, staging surgeries could identify potential retroperitoneal lymph node metastasis in patients with menopausal status, those with a tumor size >20 mm, grade 3 patients, patients with positive LVSI and those with lower uterine segment involvement. Lymphadenectomy involving PLN and/or PALN in such populations has a significant impact on survival outcomes. Therefore, for apparent stage IA endometrial carcinomas, lymphadenectomy should be performed at least in patients with larger, higher grade tumors, positive LVSI, or lower uterine involvement, if not universally. Patients who underwent a simple hysterectomy had inferior DFS compared with patients who underwent lymphadenectomy. In the univariate analysis, most of the survival results had non-significant or only marginal differences. However, after being adjusted for baseline clinicopathological characteristics and surgical modalities, these results had important significance.

Although PLN resection may increase OS (15), comprehensive nodal dissection has decreasing utility in the current treatment for endometrial cancer (16). A Cochrane review from 2017 found no evidence that lymphadenectomy for endometrial cancer decreased the risk of death or disease recurrence compared with no lymphadenectomy in women with presumed stage I disease (17). Evidence regarding serious adverse events suggests that women who undergo lymphadenectomy are more likely to experience surgery-related systemic morbidity or lymphedema and/or lymphocyst formation (17, 18). Data from the population-based Munich Cancer Registry did not show a significant benefit of systematic lymph node dissection. However, these results should be cautiously cited, since many important clinicopathological characteristics should be taken into consideration, along with the standard, individualized postsurgical treatment.

As illustrated in our study, the prevalence of metastasis to the lymph nodes was 2.1% and 3.3% in the whole population and in patients with surgery staging, respectively. Due to the low prevalence of metastasis and associated adverse events, selecting appropriate patients for systematic lymphadenectomy is essential. In our study, menopausal patients with larger, higher grade tumors, positive LVSI, and lower uterine involvement had more metastases outside of the uterus. These findings are in agreement with those of other studies. The criteria for staging were proposed by the Mayo Clinic for grade 1 or 2 endometrioid tumors (19), which were further examined through a multi-institutional evaluation (20, 21). Other pathological characteristics, such as positive pelvic nodes, lymphovascular space invasion, and myometrial invasion >50%, were suggested as key factors to direct para-aortic lymphadenectomy (22). In clinically diagnosed, early-stage endometrial cancer, increasing age is associated with an intrinsic poorer survival index regardless of lymphadenectomy and the presence of nodal metastasis (14). These studies provide practical evidence for the selection of patients eligible for lymphadenectomy.

There are debates about the application of para-aortic lymphadenectomy for staging surgeries in endometrial cancer (20, 23–25). Many surgeons do not implement a full lymphadenectomy in patients with grade 1 early-stage endometrial cancer (26). Omitting para-aortic lymphadenectomy for any grade endometrioid tumor with ≤50% myometrial invasion only missed 1.1% of para-aortic node metastasis or recurrence. Using these criteria, para-aortic lymphadenectomy may be omitted in 77% of patients with endometrioid endometrial cancer (22). However, in our study cohort, there were no significant differences in the clinicopathological characteristics of patients with and without PALN metastasis. In addition, for 22 patients with metastasis to the lymph nodes who underwent para-aortic lymphadenectomy, 5 (22.7%) had only para-aortic metastasis. These findings suggest the necessity of para-aortic lymphadenectomy in comprehensive staging surgeries when indicated.

In our study, only 66 of 836 (7.9%) patients underwent sentinel lymph node mapping, which did not reveal any benefits in the survival or surgical outcomes. Numerous studies have demonstrated that compared to systemic lymphadenectomy, sentinel lymph node mapping with ultrastaging may increase the detection of lymph node metastasis, with low false-negative rates in women with apparent uterine-confined disease and with lower morbidity (27, 28), including high-risk histology (29, 30), as well as in terms of the detection of PALN involvement (31). In a meta-analysis, the sensitivity of the overall detection rate of sentinel lymph node mapping was 96.3%, with a sensitivity of 73.1% for the bilateral sentinel node detection rate (32). Laparoscopic sentinel lymph node localization is feasible and accurately predicts the lymph node status in patients with endometrial cancer (32, 33). Utilizing indocyanine green results in favorable sentinel lymph node detection rates (33, 34). The expertise of the surgeon and attention to technical details is critical. The results of our study deserve further validation and generalization in apparent stage IA endometrial cancer patients.

The strengths of our study include its large cohort and rigorous and long-term follow-up. The limitations of our study include its retrospective design, which may cause significant observation and selection bias. LVSI was not always discovered before major surgeries for endometrial cancer, thereby limiting its utilization in the prediction of lymph node metastasis. Importantly, apparent stage IA endometrial cancer as defined in our retrospective study may produce recall bias. The main limitation of our study was the lack of a uniform preoperative imaging evaluation, since endometrial invasion cannot be accurately assessed by sonography or CT (3). The relatively small population with metastasis limited the analysis of adjuvant treatment on survival outcomes. The lack of a more detailed description of surgical outcomes and complications probably restricted the quality of life evaluation of the patients. In a report by van de Poll-Franse et al. (35), women with stage I/II endometrial cancer who underwent lymphadenectomy reported higher lymphedema symptom scores on two different quality of life questionnaires.

## Conclusions

In patients with apparent stage IA endometrial endometrioid carcinoma, lymphadenectomy could provide more information about survival outcomes, since patients with metastasis to lymph nodes had inferior DFS, OS and cancer-specific OS. Lymphadenectomy should be considered in patients of menopausal status, with larger, higher grade tumors, positive LVSI, or lower uterine segment involvement.

## Data Availability Statement

The datasets presented in this study can be found in online repositories. The names of the repository/repositories and accession number(s) can be found in the article/**Supplementary Material**.

## Ethics Statement

The studies involving human participants were reviewed and approved by The Institutional Review Board of Peking Union Medical College Hospital. The patients/participants provided their written informed consent to participate in this study.

## Author Contributions

LL conceived of the original idea for the study, interpreted the results, carried out the statistical analysis, edited the paper and is the overall guarantor. ZL obtained ethical approval, contributed to the preparation of the data set, interpreted the results and contributed to drafts of the paper. MW and JL contributed to the study design and interpretation of the results and commented on drafts of the paper. All authors contributed to the article and approved the submitted version.

## Funding

This study is supported by the CAMS Innovation Fund for Medical Sciences (CIFMS-2017-I2M-1-002). The funders had no role in the study design, data collection and analysis, decision to publish, or preparation of the manuscript.

## Conflict of Interest

The authors declare that the research was conducted in the absence of any commercial or financial relationships that could be construed as a potential conflict of interest.

## Supplementary Material

Supplementary Data Sheet 1 Raw data of all patients.
